# Towards Green Strategies of Food Security: Antibacterial Synergy of Essential Oils from *Thymus vulgaris* and *Syzygium aromaticum* to Inhibit *Escherichia coli* and *Staphylococcus aureus* Pathogenic Food Isolates

**DOI:** 10.3390/microorganisms10122446

**Published:** 2022-12-10

**Authors:** Daniela Sateriale, Giuseppina Forgione, Giuseppa Anna De Cristofaro, Serena Facchiano, Floriana Boscaino, Chiara Pagliuca, Roberta Colicchio, Paola Salvatore, Marina Paolucci, Caterina Pagliarulo

**Affiliations:** 1 Department of Science and Technology, University of Sannio, Via F. De Sanctis Snc, 82100 Benevento, Italy; 2 Institute of Food Science, National Research Council (CNR-ISA), Via Roma 64, 83100 Avellino, Italy; 3 Department of Molecular Medicine and Medical Biotechnologies, University of Naples Federico II, Via S. Pansini 5, 80131 Naples, Italy; 4 CEINGE-Biotecnologie Avanzate s.c.ar.l., Via G. Salvatore 486, 80145 Naples, Italy

**Keywords:** essential oils, antibacterial agents, antibacterial synergy, natural food preservatives, food pathogens

## Abstract

Foodborne diseases continue to represent an important public health issue. The control of food spoilage and pathogenic microorganisms is achieved mainly by synthetic chemicals, unfortunately associated to several undesirable aspects. The growing requirement for new and safe alternative strategies has resulted in the research of agents from natural sources with antimicrobial properties, such as essential oils (EOs). This study’s purpose was to define the antibacterial profile of thyme (*Thymus vulgaris*) and cloves (*Syzygium aromaticum*) essential oils against both Gram-positive and Gram-negative important foodborne pathogenic bacteria. Gas chromatography mass spectrometry analysis was performed for EOs’ chemical composition. Qualitative in vitro antimicrobial assays (i.e., agar well diffusion method and disk-volatilization method) allowed for verification of the efficacy of EOs, used individually and in binary combination and both in liquid and vapor phase, against *Staphylococcus aureus* and *Escherichia coli* food isolates. Minimal inhibitory concentrations and minimal bactericidal concentration values have been used to quantitatively measure the antibacterial activity of EOs, while the fractional inhibitory concentration index has been considered as a predictor of in vitro antibacterial synergistic effects. The microbiological tests suggest that thyme and cloves EOs, rich in bioactive compounds, are able to inhibit the growth of tested foodborne bacteria, especially in vapor phase, also with synergistic effects. Results provide evidence to consider the tested essential oils as promising sources for development of new, broad-spectrum, green food preservatives.

## 1. Introduction

The association between food consumption and infectious diseases in humans has long been recognized; Hippocrates in 460 B.C. had already argued about the strong link between human health and diet [1].

Nowadays, the issue of food safety is still one of the main public health concerns. Despite efforts and improvements in hygenic safeguards in food production techniques, foodborne pathogens still cause an alarmingly increasing number of foodborne illness outbreaks yearly all over the world [2]. The causative agents of food-borne diseases have been mainly identified in bacterial pathogens, whose control in the food processing environment is considered a major challenge. They can settle on food surfaces, grow on them and form biofilms, with a high risk for food safety [3]. Moreover, the poor hygiene of surfaces, equipment and processing environments in contact with food is a factor that significantly contributes to foodborne disease epidemics [4].

The literature has reported that contaminated food by pathogenic bacteria, such as *Escherichia coli* and *Staphylococcus aureus*, poses a serious threat to human health.

Several strains of *E. coli* are pathogenic to humans, causing intestinal and urinary tract infections, cholecystitis and septicemia [5]. Transmission of *E. coli* pathotypes, the source of foodborne diseases, can occur through ingestion of contaminated food or water by feces from infected humans or animals. Contamination of animal products often occurs during slaughter processes and meat processing. As for agricultural crops, one cause of contamination is the use of animal manure as fertilizer and contaminated water for irrigation [6]. *E. coli* also shows biofilm-producing abilities under different conditions throughout the entire food production chain, and it is known for its acid resistance mechanisms that allow the bacterium to withstand extreme conditions encountered in food processing environments, such as those related to the use of disinfectants in the fight against food pathogens [7,8].

*S. aureus* is most accountable for toxic shock syndrome, endocarditis and post-operative wound infections [9], but also for food poisonings [10]. It is possible to find *S. aureus* in many contaminated foods that include minced meat, pork sausage, minced turkey, salmon slices, oysters, shrimp, milk and salads [11]. Most cases of staphylococcal food poisoning can be traced back to food contamination during preparation due to inadequate refrigeration, inadequate cooking or heating or poor personal hygiene. There are about ten identified staphylococcal enterotoxins, but types A and D are responsible for most outbreaks [12].

The sanitary emergence of foodborne diseases quickly needs solving strategies. Food safety has now become a top priority for the global community, and there is a greater awareness of the need to improve our understanding and monitoring of foodborne diseases and pathogens, as well as to implement structured approaches in food conservation strategies.

Synthetic chemical preservatives have been commonly used in the food industry over previous decades [13]. However, there has been controversy regarding the application of these artificial chemical compounds in the control of foodborne bacteria. They have been associated with various undesirable aspects, including carcinogenicity, teratogenicity and acute toxicity [14]. Recently, due to the growing concerns regarding food safety, consumer preferences are shifting toward food products that are free of chemical additives [15]. The harmful effects associated with synthetic food preservatives have prompted a search for effective alternatives in the world of natural products [16]. Currently, several compounds are being studied that could be used as useful and safe natural food preservatives. Selected plant extracts with a wide range of antimicrobial activity are among the most promising alternatives, given their significant antibacterial and antifungal properties [17,18,19].

Essential oils (EOs), aromatic oily liquids obtained from plant material by different methods [16], could represent a better choice than some synthetic chemical additives. EOs have been used since ancient times for their preservative properties, but the scientific interest for their application in food is renewed in recent times [16]. They constitute a complex of bioactive molecules that have various interesting biological properties, such as antioxidant [20], antibacterial, antifungal and antibiofilm [21,22], allowing their use in various fields: cosmetology, medicine, pharmaceutical and food industry. EOs can be extracted from various aromatic plants, including herbs and spices [20]. Thyme (*Thymus vulgaris* L.) and clove (*Syzygium aromaticum* L.) essential oils are among the most appreciated essential oils, especially due to their significant antimicrobial properties.

*Thymus vulgaris*, commonly known as thyme, belonging to the family of *Lamiaceae*, is a flowering plant native to Southern Europe and distributed worldwide, with important pharmacological properties [23]. Thyme extracts, such as essential oil obtained from plant aerial parts (flowers and leaves), showed antibacterial properties against both Gram-negative and Gram-positive bacteria [24,25,26]. *Syzygium aromaticum* L. is an aromatic plant, belonging to the *Myrtaceae* family, commonly named clove. It is mainly cultivated in tropical and subtropical countries and is a valuable source of bioactive volatile compounds. Cloves essential oil, obtained by flower buds, has attracted considerable scientific interest thanks to its broad application in cosmetics and medicine; its antioxidant and antimicrobial activities are well documented [27]. Several studies demonstrated antibacterial effects of thyme and cloves essential oil against numerous food pathogenic isolates [28,29,30], in agreement with their potential use as natural preservatives, to produce wholesome food products, for extension of shelf-life and to reduce pathogenic bacteria.

Inappropriate and excessive use of conventional antimicrobial agents has led to the spread of multi-resistant pathogens, including foodborne, with enormous consequences for human health. Thanks to their natural antimicrobial properties, essential oils could have great potential also to remedy the growing problem of antimicrobial resistance [31]. It is, therefore, necessary to introduce new effective antimicrobial strategies to be used also in combination with traditional ones. Several evidences suggest that essential oils, even in a mixture with or as adjuvants of conventional antimicrobials, could represent effective tools against resistant pathogens [32]. Essential oils have multi-target inhibitory effects on microorganisms and can destabilize their cellular structure, thus increasing microbial susceptibility to other antimicrobial compounds [33]. The study of the antimicrobial effects of essential oils, used individually or in synergy, is therefore extremely useful.

In this context, our study aims to investigate the in vitro antibacterial properties of essential oils of thyme (*Thymus vulgaris*) and clove (*Syzygium aromaticum*) against foodborne pathogens, also in combination. The specific aim was to define the EOs antimicrobial profile against food isolates of *E. coli* and *S. aureus*, important and common causative agents of food infections. These essential oils have been tested both in liquid and in vapor phase, in order to assess the bioactivity of volatile fractions. EOs were tested also in binary combination against the food isolates in order to investigate combinatorial interactions and highlight their synergistic antimicrobial properties to be exploited in food preservation. The significance of this study is high, since the power of synergistic antibacterial activity of EO combinations, especially due to volatile antimicrobial molecules, has huge potential not yet fully explored.

## 2. Materials and Methods

### 2.1. Tested Essential Oils

Thyme essential oil (thy-EO) and cloves essential oil (cl-EO) were kindly provided by Alta Profumeria S.r.l. (Durazzano, BN, Italy), a local company in scientific collaboration with the University of Sannio for the evaluation of innovative solutions with high biocidal power and low environmental impact.

Tested EOs were obtained by conventional steam distillation method by plant aerial parts. In particular, thyme EO was obtained from both leaves and flowers of *Thyumus vulgaris* L. and cloves EO from flower buds of *Syzygium aromaticum* L. The essential oils were stored at refrigeration temperature (0–4 °C) in the dark until further use. In particular, EOs were tested within two months of being provided by the manufacturer, in compatibly with the maintenance of their bioactivity during the storage period.

### 2.2. Volatile Compound Determination by Headspace-Solid Phase Microextraction-Gaschromatography/Mass Spectrometry (HS-SPME-GC/MS)

The volatile fraction of each essential oil sample was analyzed by headspace sampling using the solid-phase microextraction technique (SPME) according to Maoloni et al. (2021) [34], with some modifications. In detail, a 50 μL aliquot of essential oil was placed in a 20 mL headspace vial. The sample was equilibrated at 40 °C for 10 min at 250 rpm using a Gerstel MPS2 automatic sampling system (Gerstel GmbH & Co., Mülheim, Germany). The analysis was conducted by a GC/MS system (Agilent 7890/5975 Inert, Agilent, Santa Clara, CA, United States) equipped with the above mentioned autosampler with helium as the carrier gas (1 mL min^−1^). A coated carboxen/polydimethylsiloxane (CAR/PDMS) fiber (Sigma Aldrich S.r.l., Milan, Italy) was exposed to the headspace of the sample for 20 min, maintaining the sample at 40 °C. The fiber was desorbed for 10 min at 240 °C in the injection unit in split mode (split ratio 50:1). The separation was carried out in a capillary column (HP-Innowax, Agilent Technologies, Santa Clara, CA, USA) (30 m × 0.25 mm i.d. × 0.50 μm film thickness). The GC oven temperature program started at 45 °C for 4 min, then was ramped to 240 °C at 4 °C min^−1^ and maintained the final temperature for 10 min. The mass spectrometer operated with an ion source of 230 °C, a quadrupole temperature of 150 °C, 70 eV electron energy and acquired in TIC mode from *m*/*z* 40–350 uma. The compounds were firstly identified based on their mass spectra using Wiley7, Nist 05 libraries. For the next identification step, a retention index (RI) was calculated for each compound according to Van Den Dool and Kratz (1963) [35] and based on a series of alkanes. The calculated RIs were then compared with Van Den Dool and Kratz RIs for high polar stationary phases using the NIST online database (http://webbook.nist.gov/chemistry/, accessed on 30 October 2022). The proportion of each compound was estimated by dividing its mean area by the total area of the chromatogram and was expressed as a percentage. All the analyses were performed in duplicate.

### 2.3. Food Bacterial Isolates and Growth Conditions

The antibacterial activity of thyme and cloves EOs was evaluated against both Gram-negative and Gram-positive bacteria, i.e., against *Escherichia coli* and *Staphylococcus aureus* food isolates, obtained from samples of minced meat chicken. Isolation and phenotypic identification of the bacterial strains by differential and chromogenic selective media were performed at the Laboratory of Microbiology of the Department of Science and Technology, University of Sannio. Food bacterial isolates were grown under aerobic conditions at a temperature of 37 °C on the non-selective medium Luria Bertani (LB) (CONDA, Madrid, Spain) and on the selective and chromogenic and differential media TBX (Tryptone Bile X-Glucuronide), chromogenic agar (CONDA, Madrid, Spain) and Baird Parker Base agar (CONDA, Madrid, Spain), with the addition of tellurite egg yolk emulsion (cat. 5129, CONDA, Madrid, Spain) for in vitro growth and identification of *E. coli* and *S. aureus* isolates, respectively.

The Gram staining (Gram staining kit, Sigma-Aldrich S.r.l., Milano, Italy) and the microscopy observation (Motic B1-223, Thermo Fisher Scientific, Waltham, MA, USA), together with oxidase (cat. MB0266, Oxoid, Hants, UK), catalase (3% hydrogen peroxide solution, Sigma-Aldrich S.r.l., Milano, Italy) and coagulase (cat. 74226, Sigma-Aldrich S.r.l., Milano, Italy) biochemical tests completed the phenotypic identification of food isolates, respectively denominated as *E. coli* mC1 and *S. aureus* mC2. Performed tests are recommended by ISO 16649-2 and ISO 6888-1, indicated for the identification of *E. coli* and *S. aureus* pathogenic bacteria in foods, respectively, in accordance with guidelines on food safety criteria within European legislation No. 2073/2005 [36]. Before use, bacterial isolates were revivified by subcultures on LB plates at 37 °C for 24 h in aerobic conditions.

### 2.4. Antibacterial Assays

#### 2.4.1. Agar Well Diffusion Method

To qualitatively evaluate the in vitro antibacterial effects of thyme and cloves EOs, also in binary combination, against food isolates, the agar well diffusion method was carried out, similarly to Perez (1990) [37], with slight modification. Briefly, foodborne isolates were sub-cultured in LB broth. The optical density (O.D.) value of 0.5 (600 nm wavelength) was reached. Then, standard aliquots of microbial inoculum were uniformly distributed on the surface of the LB agar plates with sterile swabs, and 6 mm wells were punched with sterilized glass Pasteur. Then the wells were filled up with pure EOs or EOs combination (1:1 ratio) aliquots (20, 40, 80 µL) and with positive and negative controls. In particular, gentamicin (Sigma-Aldrich S.r.l., Milano, Italy; 6000 µg/well) and vancomycin (Gold-biotechnology, Saint Louis, MI, USA; 800 µg/well) were used as positive controls for *E. coli* mC1 and *S. aureus* mC2 isolates, respectively, while distilled water was the test negative control. Incubation of plates at 37 °C was ensured for 24 h. Subsequently, the size of the inhibition zones around the wells was observed. The evaluation of in vitro antibacterial activities of tested essential oils against the selected microorganisms was carried out by measuring the mean diameter of the inhibition zones (MDIZ) (expressed in mm) produced by EOs and their combination.

#### 2.4.2. Disk-Volatilization Method

To analyze the antimicrobial activity of the volatile components of the tested EOs, the disk-volatilization method was carried out, as described by Tyagi et al. (2012) [38]. Briefly, after spreading aliquots of bacterial suspensions at 0.5 OD_600 nm_ (200 μL) on agar medium, an impregnate filter paper disc with EO, or EO binary combination (1:1 ratio) aliquots (5, 10, 20 µL), was placed in the center of the Petri plate lid. Therefore, by not placing the EOs in direct contact with the agar, it was possible to evaluate the antimicrobial activity exerted by the volatile components alone. A 5% sodium hypochlorite solution (volume of 10 µL/disk) was used as test positive control, while distilled water was used as negative control. The plates were incubated under aerobic conditions at 37 °C for 24 h. The assay results were evaluated by measuring the MDIZ produced by the volatile compounds of the essential oils against the selected microorganisms for the agar well diffusion test.

#### 2.4.3. Tube Dilution Method

The susceptibility of foodborne isolates to increasing concentrations of single EOs and EOs binary combination was determined by the tube dilution method with broth standard inoculum 1 × 10^5^ CFU mL^−1^ (Colonies Forming Units/mL), according to Clinical and Laboratory Standards Institute (CLSI) 2022 guidelines [39]. In brief, different final concentrations of pure EOs, and EOs binary combination, were obtained by adding them directly to the aqueous medium (LB broth). The vigorous stirring by a vortex mixer and the constant shaking during incubation were sufficient to obtain and maintain homogenous EOs micelle aggregates in the broth medium. This method allowed the quantitative evaluation of EOs and EOs combination antibacterial effects through the determination of minimum inhibitory concentration (MIC) and minimum bactericide concentration (MBC) values for each tested antibacterial agent. MIC values were determined thanks to incubation of bacterial cultures in the presence of EOs and EOs binary combination (1:1 ratio) at increasing concentrations (0, 2.5, 5, 10, 20, 30, 40, 60, 80, 100, 120, 160, 180, 200 μL mL^−1^), under appropriate growth conditions, with constant agitation. Then the observation of tube turbidity was performed. For MBC determination, aliquots of serial dilutions of the bacterial suspensions were spread on LB agar, and plates were incubated at 37 °C for 24 h to evaluate the viable bacterial counts. The MIC was defined as the lowest concentration of each in vitro antibacterial agent, including single EOs and EOs binary combination, which prevents bacterial growth. The MBC was assigned to the minimum concentration of each in vitro antibacterial agent that killed 99% of bacteria from the initial inoculum. Gentamicin (stock solution concentration of 30 mg mL^−1^) and vancomycin (stock solution concentration of 10 mg mL^−1^) were used as positive controls for *E. coli* mC1 and *S. aureus* mC2 isolates, respectively, while distilled water was used as negative control.

### 2.5. Determination of Vitro Synergistic Activity of Essential Oils Combination

The effects of thyme and cloves essential oils were deemed synergistic, indifferent or antagonistic against the two food pathogens, thanks to the measuring of the fractional inhibitory concentration index (FICI) of their binary combination. In particular, the following formulas, in accordance with Odds’ interpretation [40], were used. In brief, fractional inhibitory concentration (FIC) = MIC of antimicrobial agent in the binary combination/MIC of single antimicrobial agent; FICI = FIC of antimicrobial agent 1 + FIC of antimicrobial agent 2. In our study, the FIC was defined as the minimum inhibitory concentration (MIC) of the essential oil used in combination, divided by the MIC of the same oil used alone. The FICI was defined as the sum of the FICs obtained for the binary combination and expresses the type of interaction of the different agents used as in vitro antibacterial (particularly, FICI ≤ 1, synergy; FICI > 1 or ≤ 4, indifference; FICI > 4, antagonism) against each bacterial food isolate.

### 2.6. Statistical Data Analysis

All experiments were performed in triplicate, with independent microbial cultures for antimicrobial assays. The results obtained were analyzed and graphically reported by using “GraphPad Prism 7.00” software, validating the statistical significance by the one-way ANOVA test, with Dunnett’s and Tukey’s corrections, and the two-way ANOVA test, with Tukey’s correction. In all cases, *p* values < 0.05 were considered statistically significant.

## 3. Results

### 3.1. Chemical Composition of Thyme and Cloves Essential Oils

The chemical composition of thyme and cloves essential oils, performed by gas chromatography mass spectrometry analyses, is shown in Table 1. According to our results, thirty volatile compounds were identified in thyme essential oil (thy-EO), representing 99.90% of detected constituents. In particular, monoterpenic compounds constituted approximately 86.9% of thy-EO composition, with limonene (59.28 ± 0.51%) and β-pinene (10.25 ± 0.49%) as major constituents, while oxygenated terpenes accounted for 13.15%, with 13.02 ± 0.30% of thymol. A total of thirty-two volatile components representing 99.60% of the total detected constituents were identified in cloves essential oil (cl-EO). Caryophyllene (64.29 ± 0.64%), eugenol (17.00 ± 1.01%) and α-humulene (11.57 ± 0.40%) were identified as the three major constituents. The other components were present in a total amount of less than 7 %.

### 3.2. In Vitro Antibacterial Activity of Thyme and Cloves Essential Oils against Escherichia Coli and Staphylococcus Aureus Food Isolates in Liquid Phase

**Figure 1:**
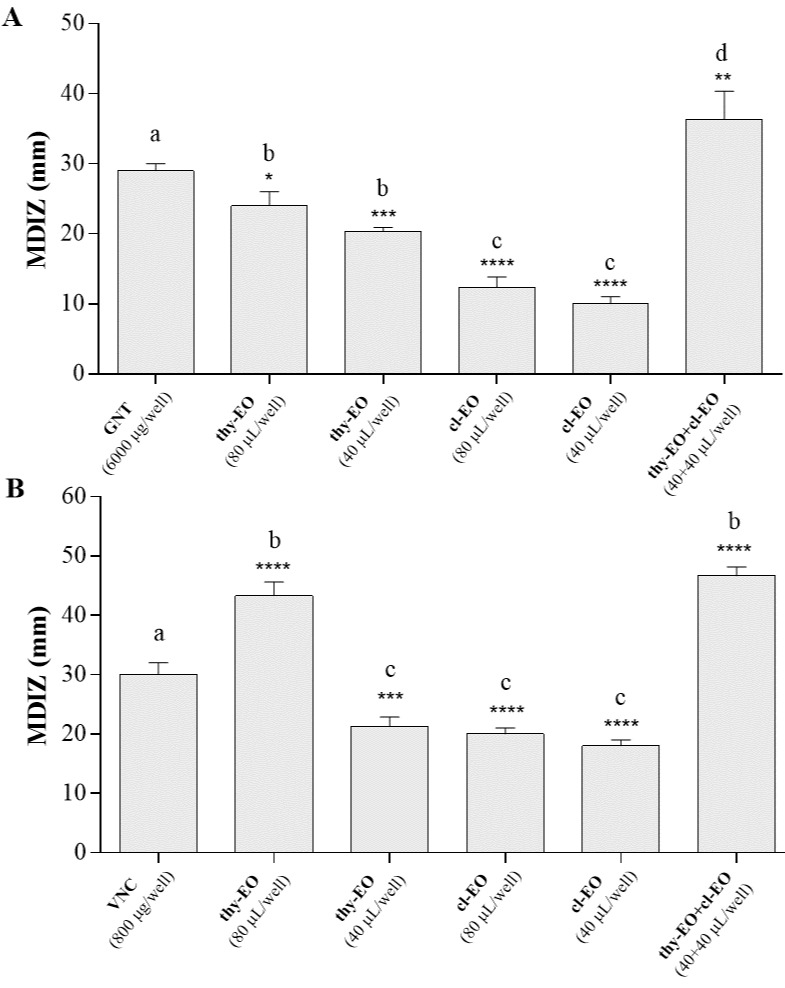
In vitro antibacterial activity of thyme and cloves essential oils, individually used and in binary combination (1:1 ratio), evaluated by the agar well diffusion method, against *Escherichia coli* (**A**) and *Staphylococcus aureus* (**B**) food isolates. Graphical representation of the results; the mean diameter of inhibition zone (in mm) is reported as the mean of values obtained from assays in triplicate ± standard deviation. Statistical significance was examined by the one-way ANOVA test, with Dunnett’s correction (*p* < 0.05) for bars comparison with positive control bar, and with Tukey’s correction (*p* < 0.05) for multiple comparisons between bars. Asterisks indicate the statistical significance respect to the positive control (**** *p* < 0.0001; *** *p* < 0.001; ** *p* < 0.01; * *p* < 0.05); the absence of asterisks indicates absence of significance. Letters (a, b, c, d) are used for multiple comparisons. Different letters indicate significant differences between bars; bars with no significant differences receive the same letter. MDIZ, mean diameter of the inhibition zone; thy-EO, thyme essential oil; cl-EO, cloves essential oil; GNT, gentamicin; VNC, vancomycin.

Thyme and cloves EOs exhibited an appreciable inhibitory activity against both *E. coli* and *S. aureus* identified foodborne isolates (Table S1), as confirmed by the values of observed inhibition zones of bacterial growth. Figure 1 shows the mean diameters of the inhibition zones (MDIZ) estimated by the agar well diffusion method for single tested EOs and their binary combination against *E. coli* mC1 (Figure 1A) and *S. aureus* mC2 (Figure 1B) isolates. The MDIZ ranged between about 10.00 ± 0.82 mm (for cloves essential oil vs *E. coli* mC1 at the volume of 40 µL/well) until about 44.67 ± 1.60 mm (observed for the binary combination of thyme essential oil and cloves essential oil vs *S. aureus* mC2 at the volume of 40 + 40 µL/well). The selected positive controls, i.e., gentamicin and vancomycin, demonstrated antibacterial effect against the isolates, with MDIZ values of 29.00 ± 0.82 mm and 30.00 ± 1.63 mm, respectively; no effects were observed for the negative control.

Quantitative assays confirmed the in vitro antibacterial activity of the EOs. Table 2 reports the values of minimum inhibitory concentration (MIC) and minimum bactericidal concentration (MBC). Thyme and cloves essential oils showed bacteriostatic and bactericidal effects against *E. coli* mC1 and *S. aureus* mC2 isolates. The inhibitory activity of EOs on food pathogens is appreciable both when they were used individually and in binary combination.

### 3.3. In Vitro Antibacterial Activity of Thyme and Cloves Essential Oils against Escherichia coli and Staphylococcus aureus Food Isolates in Vapor Phase

**Figure 2:**
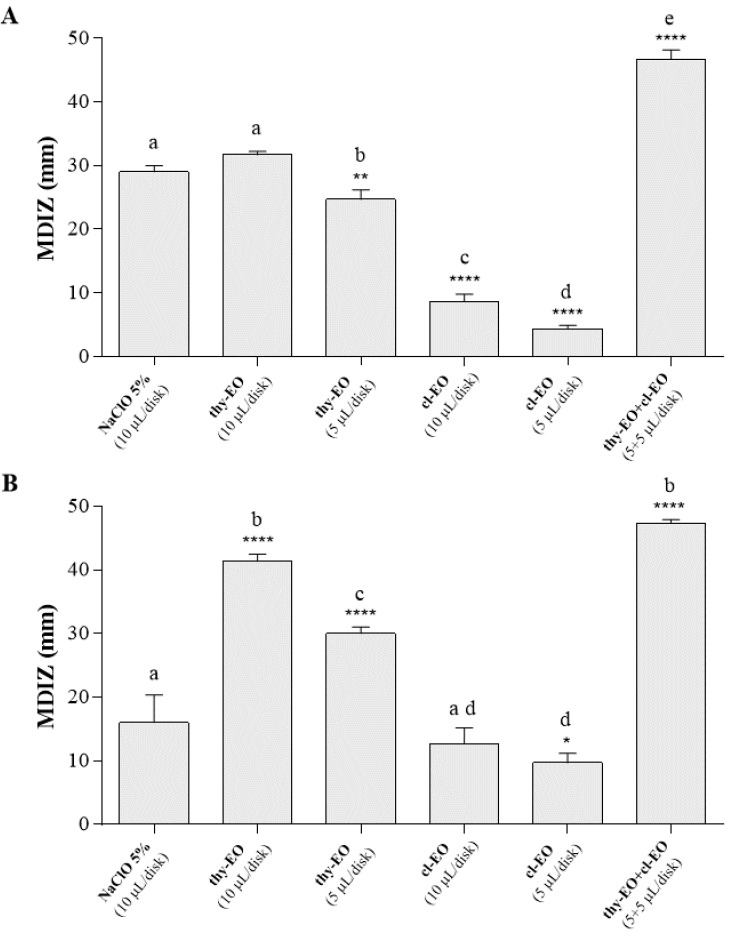
In vitro antibacterial activity of the volatile components of thyme and cloves essential oils, individually used and in binary combination (1:1 ratio), evaluated by the disk volatilization method, against *Escherichia coli* (**A**) and *Staphylococcus aureus* (**B**) food isolates. Graphical representation of the results; the mean diameter of inhibition zone (in mm) is reported as the mean of values obtained from assays in triplicate ± standard deviation. Statistical significance was examined by the one-way ANOVA test, with Dunnett’s correction (*p* < 0.05), for bars comparison with positive control bar, and with Tukey’s correction (*p* < 0.05), for multiple comparisons between bars. Asterisks indicate the statistical significance with respect to the positive control (**** *p* < 0.0001; ** *p* < 0.01; * *p* < 0.05); the absence of asterisks indicates absence of significance. Letters (a, b, c, d, e) are used for multiple comparisons. Different letters indicate significant differences between bars; bars with no significant differences receive the same letter. MDIZ, mean diameter of the inhibition zone; thy-EO, thyme essential oil; cl-EO, cloves essential oil; NaClO, sodium hypochlorite 5%.

Disk-volatilization method allowed for evaluation of the antimicrobial effects of the volatile components contained in essential oils. Results from this test (Figure 2) demonstrated the high antimicrobial effects of thyme and cloves essential oil against both *E. coli* (Figure 2A) and *S. aureus* (Figure 2B) food isolates, when tested in vapor phase. The mean diameters of the inhibition zones (MDIZ) of bacterial growth reached values up to 46.67 ± 1.25 mm (observed for the binary combination of thyme essential oil and cloves essential oil at the volume of 5 + 5 µL/disk) against *E. coli* mC1 isolate and 47.33 ± 0.47 mm (observed for the binary combination of thyme essential oil and cloves essential oil at the volume of 5 + 5 µL/disk) against *S. aureus* mC2 isolate.

**Figure 3:**
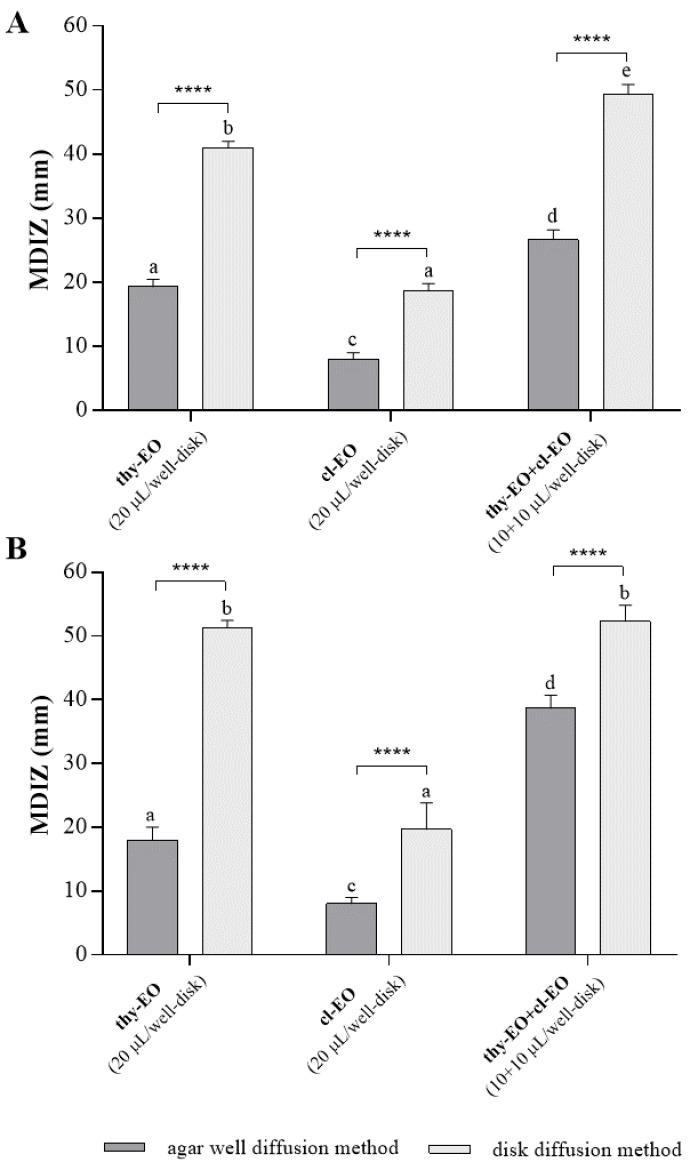
Comparative analysis of in vitro antimicrobial effects of thyme and cloves essential oils, individually used and in binary combination (1:1 ratio), evaluated with agar well diffusion method and with disk volatilization method against *Escherichia coli* (**A**) and *Staphylococcus aureus* (**B**) food isolates. The inhibition zone (in mm) is reported as mean value from triplicate assays ± standard deviation, performed using the same volume (20 µL) of each tested antibacterial agent. Statistical significance was examined by two-way ANOVA test, with Tukey’s correction (*p* < 0.05). Asterisks indicate the statistical significance between selected groups (**** *p* < 0.0001). Letters (a, b, c, d, e) are used for multiple comparisons between bars. Different letters indicate significant differences; bars with no significant differences receive the same letter. MDIZ, mean diameter of the inhibition zone. MDIZ, mean diameter of the inhibition zone; thy-EO, thyme essential oil; cl-EO, cloves essential oil.

The comparative analysis between results obtained from the agar well diffusion and the disk-volatilization methods performed at the same volume of tested EOs and EOs combination (20 µL, 10 + 10 µL) (Figure 3) showed higher antimicrobial activity of volatile components of thyme and cloves EOs in comparison to EOs tested in liquid phase against both *E. coli* mC1 (Figure 3A) and *S. aureus* mC2 (Figure 3B) food isolates. In particular, the binary combination of thyme and cloves EOs (thy-EO+cl-EO) demonstrated significantly higher antimicrobial activity in vapor phase in comparison with liquid phase against *E. coli* isolate (from 26.67 ± 1.25 mm to 49.33 ± 1.25 mm) and *S. aureus* (from 38.67 ± 1.70 mm to 52.33 ± 2.05 mm).

### 3.4. Synergistic Inhibitory Effect of Binary Combination of Thyme and Cloves EOs against E. coli and S. aureus Food Isolates

The in vitro antibacterial activity of the binary combination (1:1 ratio) of thyme and cloves essential oils has been determined by calculating the fractional inhibitory concentration (FIC) value for each EO. Subsequently, the FIC index (FICI) for the binary combination of EOs has been measured. In Table 3 are reported values of FIC and FICI. The binary combination of thyme/cloves EOs showed synergistic in vitro antibacterial effects against both *E. coli* mC1 and *S. aureus* mC2 food isolates, as shown by the FIC index (FICI) values (Table 3).

## 4. Discussion

The increasing interest in biological activities of phytocompounds has encouraged the scientific community to analyze new applications for botanical extracts, including their use as alternative antimicrobials and food preservatives.

EOs and their components show significant antimicrobial activity against different foodborne pathogens and spoilage microorganisms when tested in vitro. There are several advantages in using essential oils in food preservation. They show antimicrobial properties already at low concentrations, with no correlations with cytotoxic effects commonly associated to several synthetic additives [16]. A certain number of EOs are labelled as GRAS (generally recognized as safe), including EOs derived from cinnamon, rosemary, oregano, basil, thyme, cloves, ginger, and lavender [41] and are listed in the natural additives/preservatives admitted in the European Union Register of Feed Additives, which establishes authorized feed additives in the European market [42]. The most common tricky problem of applying EOs in food products is the maintenance of food organoleptic properties also with relatively low doses but effective against microorganisms [43]. The possible solutions proposed by previous research studies to solve these challenges include to exploit the antimicrobial power of the EOs volatile bioactive compounds to further reduce effective doses [44]. Undesirable organoleptic effects can be avoided also by using combinations of EOs [45]. The strong aroma of the EOs can affect food organoleptic quality, but the synergistic combinations of EOs with each other could decrease the total amount of EO required for the antimicrobial effect, with a consequent reduction of their impact on food organoleptic qualities [46].

The chemical composition of the essential oils of thyme and cloves employed in this study was determined by gas chromatography mass spectrometry. Even if the variance in chemical composition of volatile compounds depend on several factors, such as environment and cultivation practices [47], our results are in line with the literature. Similar studies reported thymol, α-pinene and γ-terpinene as major compounds in the thyme essential oils [48,49], with thymol percentage ranging from 12% to 71% for *Thymus vulgaris* EO [50,51]. Limonene, among the most important monoterpene hydrocarbons in essential oil from *Thymus vulgaris*, shows its maximum concentrations at the beginning of the vegetative cycle with respect to the full bloom period [52]. There are also numerous studies that reported the cloves essential oil composition. Essential oils obtained from *Syzygium aromaticum* are generally established as eugenol chemotypes, but also EOs from the same species could present different chemical composition; caryophyllene and α-humulene are among the most abundant compounds detected in cloves EOs, contributing to its biological activity [27,53]. EOs chemical composition may vary also with the extraction yield, which was about 15% both for thyme and cloves essential oils used in this study, in accordance with the literature. Previous studies demonstrated that thyme and cloves produce essential oils with a good yield, ranging from 10% to 20%, consistent with a rich content in bioactive molecules [54,55].

In this study, different in vitro antimicrobial assays allowed for verification of the ability of thyme (*Thymus vulgaris* L.) and cloves (*Syzygium aromaticum* L.) EOs, individually used and in binary combination, to effectively counteract the growth of two important foodborne pathogenic bacteria, *Escherichia coli* and *Staphylococcus aureus*, both in liquid and in vapor phases.

First, EOs’ in vitro antimicrobial activity was evaluated by the agar well-diffusion method. From this preliminary screening, it resulted that thyme essential oil (thy-EO) showed the highest antibacterial activity against both *E. coli* and *S. aureus* isolates, when individually used, with MDIZ values of 20.33 ± 0.47 mm (40 μL/well) and 24.00 ± 1.63 (80 μL/well) against *E. coli* isolate (Figure 1A) and 21.33 ± 1.25 mm (40 μL/well) and 43.33 ± 1.89 (80 μL/well) against *S. aureus* isolate (Figure 1B). Other studies also reported similar results, confirming the antibacterial activity of thyme and cloves EOs at volumes ranged between 10 μL and 100 μL [56,57]. Similarly, recent studies confirmed the significant antibacterial activity of thyme EO against *E. coli* and *S. aureus* food-borne pathogens, due to high concentrations of active compounds, such as thymol and limonene [58,59]. The binary combination of EOs (thy-EO+cl-EO) showed significant inhibitory activity against *E. coli* mC1 and *S. aureus* mC2 food isolates, as demonstrated by the mean diameter of the inhibition zone (MDIZ) of bacterial growth measured around the wells filled up with the essential oil mixture (Figure 1). Particularly, MDIZ values observed for thy-EO+cl-EO combination (40 + 40 µL/well) (36.33 ± 5.23 mm vs *E. coli* mC1 and 44.67 ± 1.60 mm vs *S. aureus* mC2) showed to be significantly major in comparison to values measured for gentamicin (29.00 ± 0.82 mm) and vancomycin (30.00 ± 1.63 mm), tested as positive controls. According to our results, Gram-positive isolate, *S. aureus* mC2, showed to be most sensitive to the two tested EOs, in accordance with literature studies, including the recent one by Alizadeh-Behbahani et al. (2019) [60], that showed the higher EOs’ antibacterial activity against Gram-positive bacteria compared to Gram-negative ones.

EOs’ in vitro antibacterial activity in liquid phase was also confirmed by quantitative antimicrobial assays. Thyme and cloves EOs showed bacteriostatic and bactericidal effects against tested bacterial strains. The MIC and MBC values did not exceed 100 μL mL^−1^ and 200 μL mL^−1^ respectively, confirming the remarkable EO antibacterial effects, especially for thyme EO. The results obtained in the present study are in agreement with El-Zehery et al. (2021) [61] and other studies [62,63], which evaluated the antibacterial activity of *Thymus vulgaris* essential oil against both Gram-negative and Gram-positive food-borne bacteria by reporting similar MIC and MBC values.

Particularly significant are the results regarding antimicrobial effects of the volatile components of essential oils. Comparing the obtained results from agar well-diffusion and disc volatilization methods, we can observe that the mean diameters of the inhibition zone are significantly higher in vapor phase with respect to the liquid one, both against *S. aureus* and *E. coli*, already with lower EOs volumes. In particular, the antimicrobial activity of thyme essential oil (thy-EO) against *E. coli* increased more than 50% (from 19.33 ± 0.94 mm to 41.00 ± 0.82 mm), while the inhibition zone values reached 51.33 ± 0.35 mm against *S. aureus*, with only 20 µL of thy-EO, the oil with the highest antimicrobial effect among those tested. Recently, several studies have confirmed that EOs in vapor phase showed a more significant bacterial inhibition effect than their liquid phase, even at lower concentrations [26,64]. The best antimicrobial effectiveness of the volatile phase compared to the liquid phase of essential oils was also reported by Laird and Phillips, (2012) [65].

The binary combination of thyme and cloves EOs showed the most remarkable inhibitory effect against food isolates, also in vapor phase. Volatile component of this oil mix led to the formation of wider inhibition zones with a mean diameter of 46.67 ± 1.25 mm against *E. coli* food isolate and 52.33 ± 2.05 against *S. aureus* isolate, compared to the MDIZ values of the positive control, i.e., the 5% sodium hypochlorite solution (27.00 ± 0.82 mm for *E. coli* mC1 and 17.55 ± 5.02 mm for *S. aureus* mC2). This binary combination showed to have synergistic antibacterial effect against both isolates, as indicated from the measured FIC index (FICI) values of 0.630 and 0.830 for *E. coli* mC1 and *S. aureus* mC2 food isolates, respectively (Table 3). In agreement with other studies, our results confirm the antimicrobial synergy of thyme and cloves EOs, maybe attributable to the synergistic effects of thymol, one of the most abundant compounds in thyme oil, which can alter membrane permeability, with eugenol of cloves oil, which can more readily reach the target proteins in the cytoplasm [66,67]. Antimicrobial mechanisms of action of EOs are heterogeneous; consequently, several in vitro studies have shown their additive or synergistic activity when EOs are used in combination [16]. This may also reduce the minimal inhibitory concentration, without changing the sensory properties of fresh food by maintaining their antimicrobial activity [68].

In conclusion, the encouraging results regarding the antibacterial and synergistic effects of thyme and cloves EOs provide evidence that they can be considered valid candidates to develop natural antimicrobial agents to control pathogen contamination in the food industry as an alternative to synthetic preservatives and, at the same time, to guarantee consumer safety. Although further studies are required to clarify mechanisms of action and synergy of EOs, these results open new perspectives for their use, mostly in mixture form, in food systems for the shelf-life improvement of perishable food products. In the near future, our scientific interest will also evaluate the biological properties of EOs encapsulated into nanoparticles or incorporated in edible/biodegradable films or coatings, as a possible solution to improve the solubility and chemical stability of phytocompounds to the advantage of their antimicrobial activity and antioxidant in food preservation [61,62].

## Figures and Tables

**Table 1:** Chemical composition of essential oils (EOs) extracted from cloves and thyme.

RI	Main Compounds	thy-EO	cl-EO
	**carbonyl compounds**		
819	2-propanone	0.02 ± 0.00	0.05 ± 0.00
1291	octanal	0.01 ± 0.00	-
1339	6-methyl-5-hepten-2-one	0.02 ± 0.00	-
1401	nonanal	0.03 ± 0.00	-
1717	citral	0.02 ± 0.00	-
	**monoterpenic compounds**		
1003	tricyclene	0.01 ± 0.00	-
1015	α-pinene	2.02 ± 0.05	0.04 ± 0.00
1019	α-tujene	0.65 ± 0.01	-
1052	camphene	0.07 ± 0.00	-
1100	β-pinene	10.25 ± 0.49	-
1114	sabinene	1.39 ± 0.08	-
1141	δ-3-carene	0.01 ± 0.00	-
1155	α-phellandrene	0.02 ± 0.00	-
1161	β-myrcene	1.48 ± 0.11	-
1173	α-terpinene	0.16 ± 0.01	-
1193	limonene	59.28 ± 0.51	0.14 ± 0.01
1203	β-phellandrene	0.81 ± 0.08	-
1235	cis ocimene	0.06 ± 0.00	-
1241	γ-terpinene	7.28 ± 0.20	-
1251	trans ocimene	0.19 ± 0.00	-
1264	o-cymene	2.13 ± 0.12	0.01 ± 0.00
1276	a-terpinolene	0.26 ± 0.02	-
1685	1,3,8-p-menthatriene	0.02 ± 0.00	-
	**sesquiterpenic compounds**		
1452	α-copaene	-	0.13 ± 0.00
1478	ylangene	-	0.02 ± 0.00
1481	α-cubebene	-	1.15 ± 0.02
1510	β-bourbonene	-	0.02 ± 0.00
1566	iso caryophyllene	-	0.18 ± 0.00
1592	caryophyllene	-	64.29 ± 0.64
1604	aromadendrene	-	0.22 ± 0.01
1660	α-humulene	-	11.57 ± 0.40
1680	α-amorphene	-	0.18 ± 0.00
1708	β-Selinene	-	0.16 ± 0.01
1715	α-selinene	-	0.13 ± 0.00
1718	α-muurolene	-	0.11 ± 0.00
1741	fernasene	-	0.05 ± 0.00
1748	δ-cadinene	-	0.88 ± 0.02
1768	curcumene	0.01 ± 0.00	0.03 ± 0.00
1824	cis calamenene	-	0.48 ± 0.02
	**oxygenated terpenes**		
1441	cis limonene oxide	0.08 ± 0.00	-
1972	caryophyllene oxide	-	0.06 ± 0.00
2189	thymol	13.02 ± 0.30	-
2225	carvacrol	0.02 ± 0.00	0.16 ± 0.01
2187	eugenol	0.02 ± 0.00	17.00 ± 1.01
2263	eugenyl acetate	-	0.75 ± 0.00
2356	isoeugenol	-	0.19 ± 0.00
	**others**		
900	dichloromethane	0.03 ± 0.00	0.08 ± 0.00
909	ethanol	0.54 ± 0.07	0.91 ± 0.04
1614	1,2-propanediol	-	0.60 ± 0.01
	***Total identified***	***99.9%***	***99.6%***
	*carbonyl compounds*	*0.09%*	*0.05%*
	*monoterpenic compounds*	*86.09%*	*0.19%*
	*sesquiterpenic compounds*	*0.01%*	*79.61%*
	*oxygenated terpenes*	*13.15%*	*18.17%*
	*others*	*0.57%*	*1.59%*

Results are reported as A% = (area peak compound/area peak total compounds) × 100 (A% ± SD). The calculated retention indices were compared with Van Den Dool RIs (polar column) for InnoWAX or similar stationary phases using online NIST database (http://webbook.nist.gov/chemistry/, accessed on 30 October 2022). RI, retention index; thy-EO, thyme essential oil; cl-EO, cloves essential oil; -, not detected.

**Table 2:** Quantitative evaluation of in vitro antibacterial activity of thyme and cloves essential oils, individually used and in binary combination, against *Escherichia coli* and *Staphylococcus aureus* food isolates.

Antibacterial Agent	*E. coli* mC1		*S. aureus* mC2	
	MIC	MBC	MIC	MBC
**thy-EO**	20 µL mL^−1^	100 µL mL^−1^	20 µL mL^−1^	80 µL mL^−1^
**cl-EO**	100 µL mL^−1^	200 µL mL^−1^	80 µL mL^−1^	100 µL mL^−1^
**thy-EO + cl-EO (1:1 ratio)**	10 µL mL^−1^	80 µL mL^−1^	10 µL mL^−1^	80 µL mL^−1^
**GNT**	50 μg mL^−1^	500 μg mL^−1^	-	-
**VNC**	-	-	100 μg mL^−1^	400 μg mL^−1^

MIC, minimum inhibitory concentration; MBC, minimum bactericidal concentration; thy-EO, thyme essential oil; cl-EO, cloves essential oil; GNT, gentamicin; VNC, vancomycin.

**Table 3:** Synergistic antibacterial effects of thyme and cloves essential oils in binary combination against *Escherichia coli* and *Staphylococcus aureus* food isolates.

Bacterial Isolate	Binary Combinations	Individual FIC	FIC Index (FICI)	Interaction Interpretation
***E. coli* mC1**	thy-EO + cl-EO	0.500–0.130	0.630	**synergy**
***S. aureus* mC2**	thy-EO + cl-EO	0.500–0.330	0.830	**synergy**

Thy-EO, thyme essential oil; cl-EO, cloves essential oil; FIC, fractional inhibitory concentration, FICI, fractional inhibitory concentration index; FICI ≤ 1.00, synergy; 1.00 < FICI ≤ 4.00, indifference; FICI > 4.00, antagonism.

## Data Availability

Not applicable.

## Supplementary Materials

The following supporting information can be downloaded at: https://www.mdpi.com/article/10.3390/microorganisms10122446/s1, Table S1: Morphological characteristics of bacterial cells and results of biochemical tests performed on food isolates.
