# Supplementary material for: Towards Green Strategies of Food Security: Antibacterial Synergy of Essential Oils from Thymus vulgaris and Syzygium aromaticum to Inhibit Escherichia coli and Staphylococcus aureus Pathogenic Food Isolates
Source: Microorganisms. 2022 Dec 10;10(12):2446. doi: 10.3390/microorganisms10122446 (PMC9780947; doi:10.3390/microorganisms10122446)
Supplement: Supplementary file 1 [file microorganisms-10-02446-s001.zip › microorganisms-2034852-supplementary.pdf]

Table S1: Morphological characteristics of bacterial cells and results of biochemical tests performed on food isolates.

Bacterial isolate	Gram staining	Oxidase test	Catalase test	Coagulase test
E. coli mC1	- (rod-shaped, 1.50x0.50 μm)	-	+	-
S. aureus mC2	+ (grape clusters spherical-shaped, 0.5 μm)	-	+	+

Legend: + = positive test; - = negative test
